# Joint line obliquity change was associated with femoral and tibial resection patterns and smaller final flexion imbalance after robot‐assisted total knee arthroplasty

**DOI:** 10.1002/jeo2.70936

**Published:** 2026-10-03

**Authors:** Yugo Morita, Jonggu Shin, Mohammed El‐Hassan, Ittai Shichman, William J. Long, Peter K. Sculco

**Affiliations:** ^1^ Complex Joint Reconstruction Center, Adult Reconstruction and Joint Replacement Service Hospital for Special Surgery New York New York USA

**Keywords:** arithmetic hip–knee–ankle angle, coronal plane alignment of the knee, intraoperative balance, joint line obliquity, robot‐assisted total knee arthroplasty

## Abstract

**Purpose:**

Associations between preoperative‐to‐post‐operative changes in arithmetic hip–knee–ankle angle (aHKA) and joint line obliquity (JLO) and final balance after robot‐assisted total knee arthroplasty (TKA) were evaluated. Relationships with resection redistribution and tibial‐varus reduction were also examined.

**Methods:**

A retrospective single‐surgeon series of 402 primary robot‐assisted TKAs was analysed. Preoperative and post‐operative aHKA, JLO and coronal plane alignment of the knee (CPAK) type were determined from full‐length radiographs. Final extension and flexion imbalance and laxity ranges after trial implantation were the primary outcomes. Signed and absolute changes were assessed in multivariable models; resection redistribution and a varus‐only tibial‐boundary subgroup were examined in supportive analyses.

**Results:**

A change in CPAK type was observed in 300 knees (74.6%), including a JLO‐zone change in 221 (55.0%). Greater ΔJLO was associated with smaller final flexion imbalance (β = −0.060; p < 0.001) and a narrower final extension laxity range (*β* = −0.055; *p* = 0.002). Corresponding associations were found for |ΔJLO| (*β* = −0.046; *p* = 0.018 and *β* = −0.070; *p* = 0.004). Neither signed nor absolute aHKA change was associated with final extension or flexion imbalance. In varus knees, tibial‐varus reduction was associated with greater JLO and aHKA changes, coronal femoral adjustment and smaller final flexion imbalance. Neither |ΔaHKA| nor |ΔJLO| was associated with improvement in the Knee injury and Osteoarthritis Outcome Score for Joint Replacement.

**Conclusions:**

Greater JLO change was associated with smaller final flexion imbalance and a narrower extension laxity range rather than impaired balance. In varus knees, JLO change accompanied tibial‐varus reduction and coordinated femoral adjustment. JLO change may therefore reflect specific aspects of the final balancing state rather than an unintended deviation from the native phenotype.

**Level of Evidence:**

Level III, retrospective cohort study.

**Abbreviations**
aHKA: arithmetic hip–knee–ankle angle
BMI: body mass index
CI: confidence interval
CPAK: coronal plane alignment of the knee
CR: cruciate‐retaining
HKA: hip–knee–ankle angle
ICC: intraclass correlation coefficient
IRB: institutional review board
JLO: joint line obliquity
KL: Kellgren–Lawrence
KOOS‐JR: Knee injury and Osteoarthritis Outcome Score for Joint Replacement
LDFA: lateral distal femoral angle
MCID: minimal clinically important difference
MPTA: medial proximal tibial angle
PCL: posterior cruciate ligament
PROMs: patient‐reported outcome measures
PS: posterior‐stabilised
SD: standard deviation
SMD: standardised mean difference
TKA: total knee arthroplasty

## INTRODUCTION

Quantitative coronal alignment planning and intraoperative assessment of extension and flexion gaps and coronal laxity are enabled by robot‐assisted total knee arthroplasty (TKA) [3, 6, 22]. Post‐operative coronal phenotype is determined by native morphology and surgical adjustments, including bone resection, implant positioning and soft‐tissue balancing; consequently, it should be interpreted in the context of the balancing process [20].

Overall limb alignment and joint line obliquity (JLO) are combined in the coronal plane alignment of the knee (CPAK) classification to describe coronal knee phenotype [20]. Inconsistent associations between CPAK type or change, JLO preservation and clinical outcomes have been reported [1, 4, 5, 8, 16, 17]. The limited ability of CPAK, arithmetic hip–knee–ankle angle (aHKA) and JLO to predict tibiofemoral gaps has also been demonstrated [14], whereas JLO preservation and predefined gap targets were achieved in most knees in a prospective robotic anatomo‐functional alignment series [9]. These observations suggest that radiographic phenotype and soft‐tissue balance should not be interpreted in isolation.

Distinct implant‐positioning and resection adjustments have been documented for varus and valgus knees treated with robotic functional alignment [18], and patient‐specific laxity phenotype patterns have been shown to be altered by bony alignment decisions [12]. Preoperative‐to‐post‐operative changes in CPAK or JLO are readily identified on serial full‐length radiographs and may be interpreted as unintended deviation from the native phenotype. However, comparable alignment and balance goals can be achieved through different combinations of femoral and tibial adjustments within a functional alignment strategy. Characterising how changes in aHKA and JLO are associated with final gaps, laxity and resection redistribution may therefore help surgeons interpret post‐operative phenotype change within the balancing workflow rather than as an isolated technical error. This information is not intended to define a universal radiographic target or preferred adjustment sequence. However, the relationship between CPAK change and the final intraoperative balance achieved during robot‐assisted TKA remains unclear.

From a surgical perspective, this distinction is relevant because restoring native JLO retains the patient's coronal joint‐line orientation, whereas accepting some change in JLO may provide flexibility for component adjustments intended to achieve acceptable gaps within predefined alignment boundaries. Previous studies have shown that larger aHKA correction may be associated with residual lateral laxity without corresponding differences in patient‐reported outcomes and that functional alignment can achieve reproducible intraoperative balance, underscoring that radiographic phenotype, balance, and clinical outcomes do not necessarily coincide [13, 28]. Evaluating post‐operative JLO change in relation to final intraoperative balance may therefore help surgeons interpret post‐operative phenotype transitions in the context of the achieved balance, while the patient‐level consequences of JLO preservation or alteration remain uncertain.

The primary objective of this study was to determine whether preoperative‐to‐post‐operative changes in aHKA and JLO were associated with final intraoperative balance after robot‐assisted TKA. Secondary objectives were to determine whether JLO change was associated with compartment‐specific resection redistribution, whether tibial‐varus reduction in varus knees was associated with CPAK/JLO redistribution and final intraoperative balance, and whether absolute changes in aHKA and JLO were associated with improvement in patient‐reported outcomes.

## MATERIALS AND METHODS

### Study design and cohort

A consecutive series of primary robot‐assisted TKAs performed by a single surgeon for osteoarthritis between August 2022 and March 2025 was retrospectively reviewed. Of 470 primary robot‐assisted TKAs, 24 knees were excluded because of rotating‐hinge implants (*n* = 6), post‐traumatic deformity (*n* = 10), or missing preoperative and/or post‐operative radiographs required for radiographic alignment assessment (*n* = 8). Of the remaining 446 eligible knees, 44 were excluded because complete paired initial and final intraoperative balance measurements were unavailable. The final analytic cohort comprised 402 knees in 355 patients (Figure 1).

**Figure 1:**
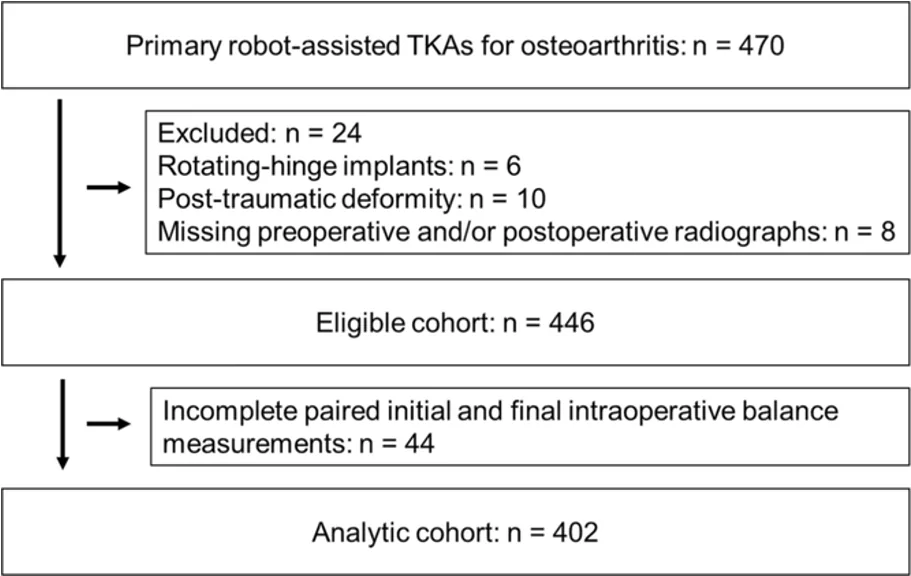
Study flow diagram. Among 470 primary robot‐assisted total knee arthroplasties performed for osteoarthritis, 24 knees were excluded because of rotating‐hinge implants (*n* = 6), post‐traumatic deformity (*n* = 10) or missing preoperative and/or post‐operative radiographs required for radiographic alignment assessment (*n* = 8). Of the remaining 446 eligible knees, 44 were excluded because paired initial and/or final intraoperative balance measurements were unavailable. The final analytic cohort included 402 knees. TKA, total knee arthroplasty.

### Surgical technique and intraoperative balance measurements

All TKAs were performed through a medial parapatellar approach with an imageless ROSA Knee System (Zimmer Biomet) workflow. Component planning was individualised according to intraoperative laxity data and deformity pattern. Restricted kinematic alignment was used for varus knees [27] within predefined coronal boundaries: an intended tibial boundary of approximately 3° of varus, a femoral coronal boundary from 2° of valgus to 1° of varus, and an overall hip–knee–ankle angle (HKA) target generally between 2° of valgus and 3° of varus. Greater residual varus was accepted selectively in knees with marked constitutional varus, although residual HKA was generally kept below 4° of varus. Within these boundaries, tibial varus was either maintained or reduced towards a more neutral obliquity according to the intraoperative gap and laxity profile. A femoral‐referenced slight valgus mechanical alignment strategy was used for valgus knees [29], with an intended post‐operative HKA target between neutral alignment and 1.5° of residual valgus when compatible with the intraoperative gap and laxity profile. Tibial slope was set to approximate the native slope, up to 8°.

After landmark registration, manual varus and valgus stress was applied by the same operating surgeon using a consistent technique with the knee in extension and at 90° of flexion, and medial and lateral compartment gaps and coronal angular excursion were recorded by the robotic platform [25, 26]. The applied force was not standardised to a predefined magnitude, and no instrumented tensioning device was used. Strictly symmetric gap targets were not required. Under the functional alignment protocol, the medial compartment was generally targeted to be approximately 1 mm tighter than the lateral compartment in extension, and the medial compartment in flexion was generally targeted to be approximately 1 mm tighter than the medial compartment in extension. These targets were applied across most preoperative alignment patterns, although symmetric gaps were accepted in severe valgus knees when soft‐tissue release was required. Component position was iteratively adjusted within the planned alignment strategy according to the measured gap and laxity profile.

Distal femoral and proximal tibial resections were performed under robotic guidance, and extension balance was reassessed after these initial resections. Selective soft‐tissue release or resection‐level adjustment was performed when required to achieve acceptable extension balance. Femoral component rotation and posterior femoral resection were then guided by flexion balance at 90°. After femoral preparation, posterior osteophytes were removed, and a limited posterior capsular release was performed when necessary. Trial components were inserted, and balance was reassessed in extension and at 90° of flexion before final implantation. After completion of the planned bone resections, distal femoral, posterior femoral and proximal tibial resection thicknesses were verified intraoperatively using the dedicated ROSA resection verification device and recorded by the robotic platform. Tibial construct thickness was defined as the combined tibial baseplate and polyethylene thickness used at the final robotic assessment. Implant design, either cruciate‐retaining (CR) or posterior‐stabilised (PS), was selected intraoperatively according to ligamentous stability and balancing requirements. In CR knees, the posterior cruciate ligament was preserved and selectively released only when required by flexion balance, whereas it was resected in PS knees.

Robotic measurements from two standardised time points were analysed: the initial assessment and the final assessment after completion of balancing and trial implantation [24]. Absolute imbalance was defined as the absolute medial–lateral gap difference. Varus–valgus laxity range was defined as the maximum varus angle minus the maximum valgus angle [3].

### Radiographic measurements and phenotype definitions

Medial proximal tibial angle (MPTA) and lateral distal femoral angle (LDFA) were measured using standard definitions on standing full‐length radiographs obtained preoperatively and at 6 weeks post‐operatively [21]. All measurements were performed by one observer. Measurements in 50 randomly selected knees were independently repeated by a second observer to assess interobserver reliability, and the primary observer repeated the measurements in the same knees to assess intraobserver reliability. Reliability was assessed using two‐way, absolute‐agreement, single‐measure intraclass correlation coefficients (ICCs). Interobserver agreement was good to excellent for LDFA (ICC, 0.855) and MPTA (ICC, 0.895), whereas intraobserver agreement was excellent for LDFA (ICC, 0.949; 95% confidence interval [CI], 0.911–0.971) and MPTA (ICC, 0.948; 95% CI, 0.911–0.970).

Coronal phenotype was characterised using aHKA and JLO. The aHKA was calculated as MPTA minus LDFA, such that varus values were negative and valgus values were positive [20]. JLO was calculated as MPTA plus LDFA, with 180° indicating a neutral joint line. Post‐operative changes in aHKA and JLO were defined as the principal radiographic exposures. Because varus aHKA values were negative and valgus values were positive, a positive ΔaHKA represented a more valgus‐directed correction.

For descriptive purposes, knees were also classified according to the original nine‐type CPAK classification. For subgroup analyses, varus knees were defined by a preoperative aHKA less than −2° and valgus knees by a preoperative aHKA greater than 2°. Neutral knees were excluded from deformity‐specific subgroup analyses.

### Statistical analysis

Continuous data were presented as mean ± standard deviation (SD) and range, and categorical data as counts and percentages. The included cohort and otherwise eligible knees excluded because paired robotic measurements were unavailable were descriptively compared using absolute standardised mean differences (SMDs) to assess potential selection bias. Final extension imbalance, final flexion imbalance, final extension laxity range and final flexion laxity range were evaluated as the main outcomes. Final flexion imbalance greater than 2.5 mm was evaluated as a secondary binary outcome [15]. Signed and absolute changes in aHKA and JLO were evaluated separately. Final‐outcome models were adjusted for the corresponding initial balance or laxity measure, age, sex, body mass index (BMI) and implant type. Patient‐level cluster‐robust standard errors were used throughout. To facilitate interpretation of effect magnitude, both the continuous exposure and outcome were standardised within each model‐specific analytic sample, and standardised *β* coefficients were calculated. Unstandardised estimates were also expressed per 5° change in each radiographic exposure.

Associations between preoperative and post‐operative aHKA and JLO and between preoperative deformity magnitude and post‐operative change were examined in radiographic redistribution analyses. Neutral knees were excluded from varus and valgus subgroup analyses. Associations of aHKA and JLO changes with compartment‐specific resection thicknesses and tibial construct thickness were evaluated in supportive exploratory analyses.

In a separate varus‐only tibial‐boundary analysis, knees were classified as native‐matched when post‐operative tibial varus was restored within 1° of native tibial varus, as tibial‐varus‐reduction when post‐operative tibial varus was reduced by more than 2° from native, or as intermediate otherwise. Additional exploratory models were used to evaluate whether tibial‐varus reduction was associated with coronal femoral adjustment, including change in LDFA and distal femoral resection asymmetry, and whether these variables attenuated its association with final flexion imbalance.

The Knee injury and Osteoarthritis Outcome Score for Joint Replacement (KOOS‐JR), a validated short‐form knee arthroplasty outcome measure, was assessed preoperatively and at 1 year post‐operatively. Improvement in KOOS‐JR and achievement of the minimal clinically important difference (MCID) were evaluated in exploratory analyses. MCID achievement was defined as an improvement of at least 16 points, based on a 1‐year Patient Acceptable Symptom State–anchored KOOS‐JR threshold reported by Dekhne et al. [10, 19]. Models were adjusted for age, sex, BMI, implant type, absolute preoperative aHKA and Kellgren–Lawrence (KL) grade. Because this retrospective study was designed to characterise patterns of association across related radiographic and intraoperative balance measures rather than to test a single confirmatory hypothesis, no formal adjustment for multiple comparisons was applied. The four main balance outcomes were distinguished from the radiographic redistribution, secondary, subgroup, resection‐related, and KOOS‐JR analyses, which were considered exploratory. Results from the latter analyses were interpreted as hypothesis‐generating. All statistical analyses were performed using R version 4.4.2 (R Foundation for Statistical Computing).

### Ethical approval

Institutional review board approval (IRB 2025‐0624) was obtained from the participating institution, and informed consent was waived owing to the retrospective nature of the study.

## RESULTS

### Radiographic characteristics and descriptive phenotype change

The mean age at surgery was 69.7 ± 8.3 years, and the mean BMI was 31.4 ± 6.1 kg/m^2^; 247 knees (61.4%) were from female patients (Table 1). Preoperative and post‐operative radiographic and intraoperative balance characteristics were summarised in Table 1. The mean preoperative aHKA was −1.3 ± 5.7°, and the mean post‐operative aHKA was −2.2 ± 3.1°. The mean preoperative JLO was 174.8 ± 3.4°, and the mean post‐operative JLO was 178.1 ± 2.7°. A change in overall CPAK type was observed in 300 knees (74.6%), including a coronal‐zone change in 185 knees (46.0%) and a JLO‐zone change in 221 knees (55.0%) (Figure 2). The most frequent phenotype‐specific transitions were from Type I to Type IV (63/144 knees, 43.8%), Type III to Type V (37/84, 44.0%), Type II to Type V (24/68, 35.3%), Type I to Type V (22/144, 15.3%) and Type VI to Type V (21/37, 56.8%) (Figure 3 and Table S4).

**Table 1:** Cohort characteristics, radiographic alignment variables, intraoperative balance measurements and CPAK change in the analytic cohort.

Variable	Analytic cohort, *n* = 402
Age, years	69.7 ± 8.3 (range, 40.0–90.0)
Sex, women, *n* (%)	247 (61.4%)
BMI, kg/m^2^	31.4 ± 6.1 (range, 16.4–53.4)
Preoperative MPTA (°)	86.8 ± 3.4 (range, 77.6–96.8)
Preoperative LDFA (°)	88.0 ± 3.2 (range, 77.1–96.7)
Post‐operative MPTA (°)	87.9 ± 2.1 (range, 81.9–93.6)
Post‐operative LDFA (°)	90.2 ± 2.0 (range, 83.3–97.2)
Preoperative aHKA (°)	−1.3 ± 5.7 (range, −15.2–19.7)
Post‐operative aHKA (°)	−2.2 ± 3.1 (range, −11.7–5.1)
Change in aHKA (°)	−1.0 ± 4.4 (range, −20.0–11.2)
Preoperative JLO (°)	174.8 ± 3.4 (range, 161.8–184.1)
Post‐operative JLO (°)	178.1 ± 2.7 (range, 167.2–186.9)
Change in JLO (°)	3.3 ± 4.1 (range, −9.5–15.3)
Initial extension imbalance (mm)	3.1 ± 2.4 (range, 0.0–14.3)
Final extension imbalance (mm)	1.1 ± 1.1 (range, 0.0–10.0)
Initial flexion imbalance (mm)	3.5 ± 2.8 (range, 0.0–18.3)
Final flexion imbalance (mm)	1.3 ± 1.4 (range, 0.0–8.3)
Initial varus‐valgus range (extension) (°)	10.5 ± 2.7 (range, 5.2–22.8)
Final varus‐valgus range (extension) (°)	4.4 ± 1.5 (range, 0.7–11.7)
Initial varus‐valgus range (flexion) (°)	9.6 ± 6.4 (range, 0.0–26.0)
Final varus‐valgus range (flexion) (°)	5.0 ± 3.6 (range, 0.0–14.6)
Overall CPAK changed, n (%)	300 (74.6%)
Coronal‐zone CPAK changed, n (%)	185 (46.0%)
JLO‐zone CPAK changed, n (%)	221 (55.0%)
Implant type, *n* (%)	
CR	353 (87.8%)
PS	49 (12.2%)

*Note*: Values are presented as mean ± SD (range) or *n* (%) unless otherwise indicated. The aHKA was calculated as MPTA minus LDFA, with varus negative and valgus positive. JLO was calculated as MPTA plus LDFA, with 180° indicating a neutral joint line. Imbalance was defined as the absolute difference between the medial and lateral spaces. Varus–valgus range was defined as the maximum varus angle minus the maximum valgus angle.

Abbreviations: aHKA, arithmetic hip–knee–ankle angle; BMI, body mass index; CPAK, Coronal Plane Alignment of the Knee; CR, cruciate‐retaining; JLO, joint line obliquity; LDFA, lateral distal femoral angle; MPTA, medial proximal tibial angle; PS, posterior‐stabilised; SD, standard deviation.

**Figure 2:**
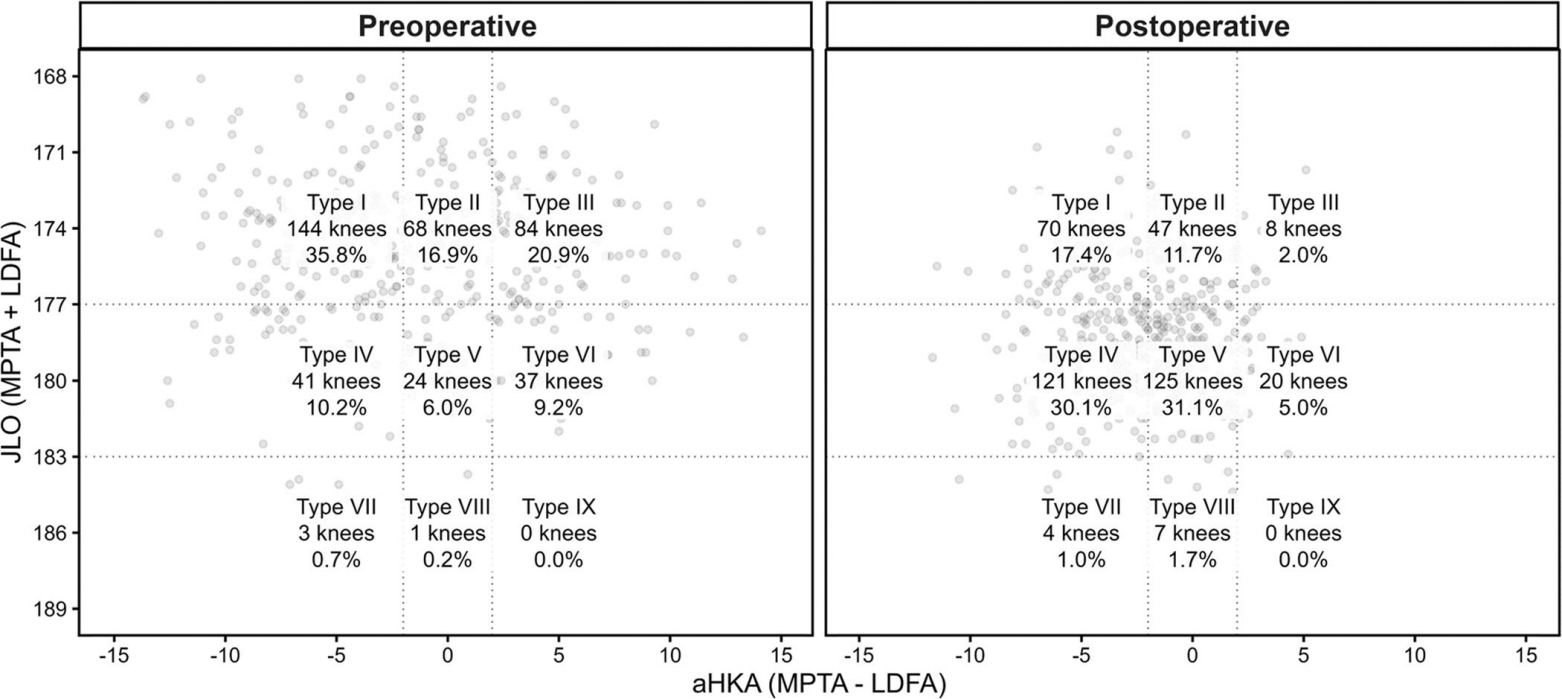
Preoperative and post‐operative distribution of CPAK phenotypes. Scatterplots show the distribution of knees according to arithmetic hip–knee–ankle angle (aHKA) and joint line obliquity (JLO) before and after surgery in the analytic cohort. Each point represents one knee. Dotted vertical lines indicate the aHKA thresholds at −2° and 2°, and dotted horizontal lines indicate the JLO thresholds at 177° and 183°, defining the original nine‐type CPAK classification. Labels within each cell show the corresponding CPAK type, number of knees and percentage of the cohort. The aHKA was defined as MPTA minus LDFA, and JLO as MPTA plus LDFA. CPAK, coronal plane alignment of the knee; LDFA, lateral distal femoral angle; MPTA, medial proximal tibial angle.

**Figure 3:**
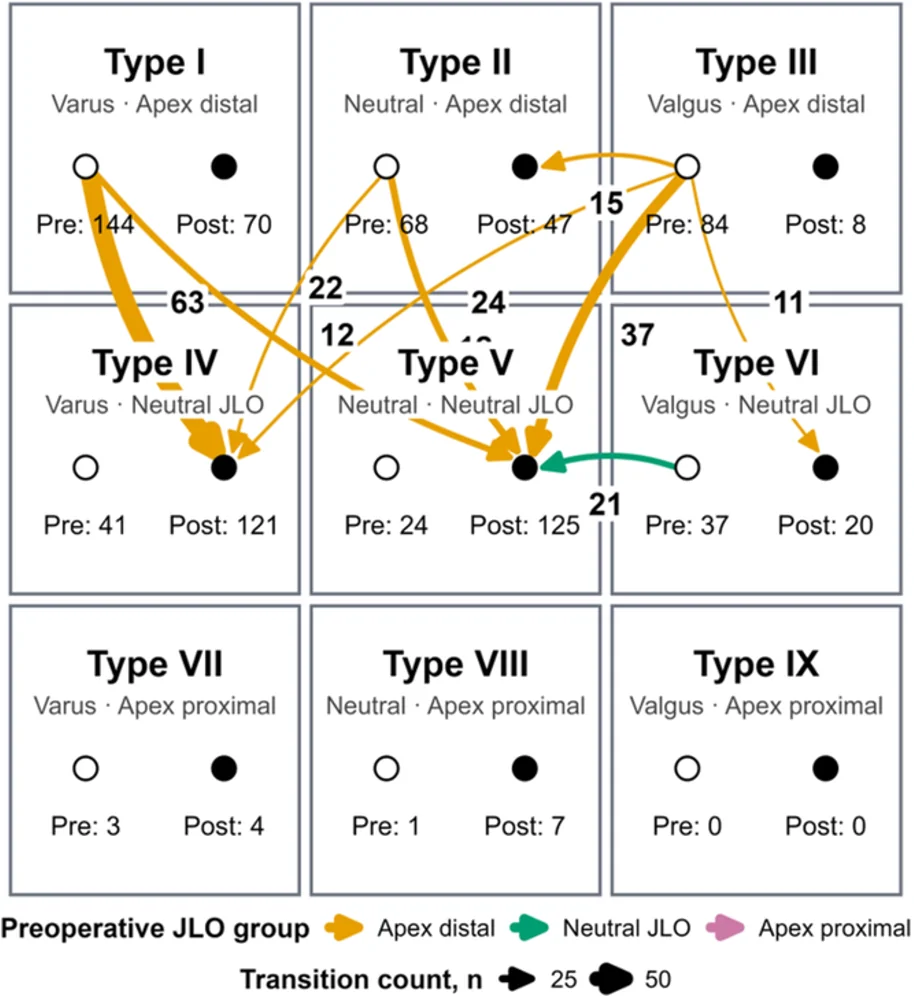
Transitions between preoperative and post‐operative CPAK phenotypes. The nine CPAK phenotypes are arranged according to the aHKA and JLO categories. Each cell shows the preoperative and post‐operative numbers of knees. Curved arrows indicate off‐diagonal transitions involving at least 10 knees; arrow width is proportional to the number of knees, and adjacent labels indicate the transition count. The complete 9 × 9 transition matrix is provided in Table S4. aHKA, arithmetic hip–knee–ankle angle; CPAK, coronal plane alignment of the knee; JLO, joint line obliquity.

### Post‐operative radiographic redistribution analysis

Moderation, but not elimination, of preoperative coronal deformity was observed post‐operatively (Figure 4). Post‐operative aHKA remained associated with preoperative aHKA (*β* = 0.319; *p* < 0.001), and greater absolute preoperative aHKA was associated with greater absolute change in aHKA (*β* = 0.480; *p* < 0.001) and movement towards coronal neutralisation (odds ratio, 1.841; *p* < 0.001). No significant association was identified between post‐operative and preoperative JLO (*p* = 0.082), whereas greater preoperative JLO deviation from 180° was associated with greater absolute change in JLO (*β* = 0.691; *p* < 0.001).

**Figure 4:**
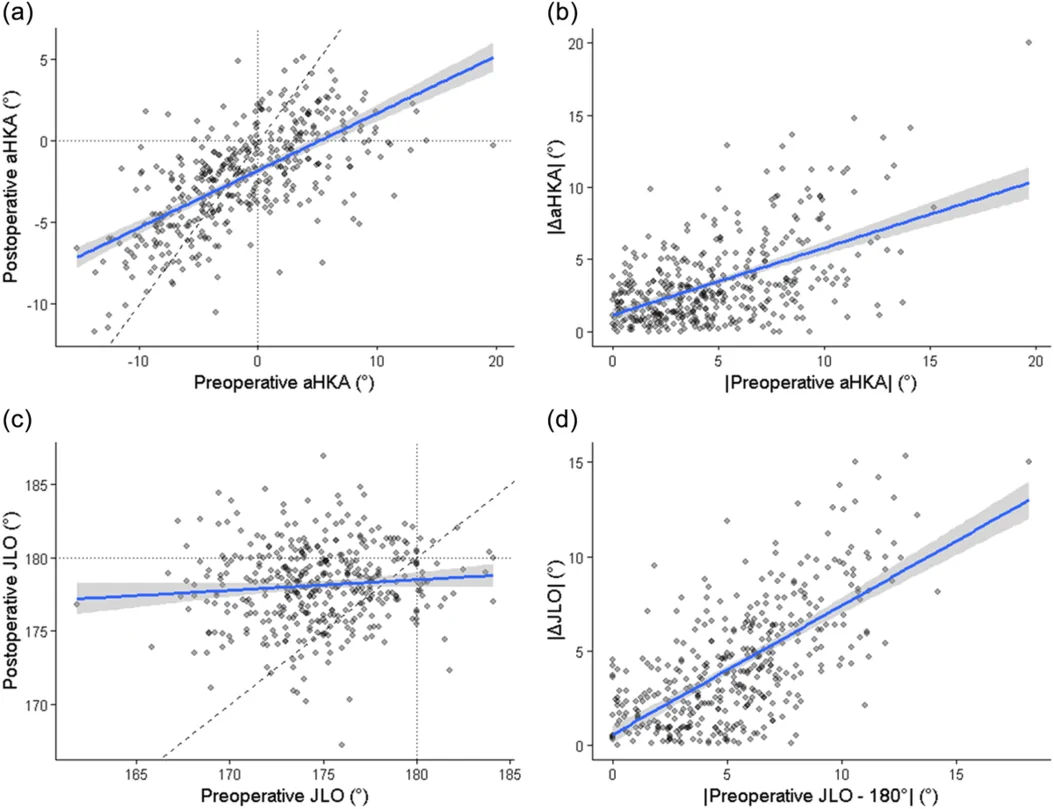
Post‐operative radiographic redistribution of coronal alignment and joint line obliquity (JLO). (a) Association between preoperative and post‐operative arithmetic hip–knee–ankle angle (aHKA). The dashed diagonal line indicates the line of identity, and the dotted horizontal and vertical lines indicate neutral coronal alignment at 0°. (b) Association between the magnitude of preoperative coronal deformity and the magnitude of post‐operative coronal alignment change, expressed as absolute preoperative aHKA and absolute change in aHKA, respectively. (c) Association between preoperative and post‐operative JLO. The dashed diagonal line indicates the line of identity, and the dotted horizontal and vertical lines indicate neutral JLO at 180°. (d) Association between the magnitude of preoperative deviation in JLO from neutral and the magnitude of post‐operative JLO change, expressed as absolute deviation of preoperative JLO from 180° and absolute change in JLO, respectively. Each point represents one knee. Solid blue lines indicate fitted linear regression lines, with shaded areas representing 95% confidence intervals. The aHKA was defined as MPTA minus LDFA, and JLO as MPTA plus LDFA. MPTA, medial proximal tibial angle; LDFA, lateral distal femoral angle.

### Associations with final balance outcomes in the overall cohort

Greater ΔJLO was associated with smaller final flexion imbalance (unstandardised *β* = −0.060; standardised *β* = −0.182; *p* < 0.001) and a narrower final extension laxity range (unstandardised *β* = −0.055; standardised *β* = −0.151; *p* = 0.002) (Table 2). Expressed over a 5° increase in ΔJLO, these estimates corresponded to a 0.301‐mm smaller final flexion imbalance and a 0.273° narrower final extension laxity range. Corresponding associations were observed for greater |ΔJLO| with final flexion imbalance (unstandardised *β* = −0.046; standardised *β* = −0.106; *p* = 0.018) and final extension laxity range (unstandardised *β* = −0.070; standardised *β* = −0.150; *p* = 0.004). In contrast, neither ΔaHKA nor |ΔaHKA| was associated with final absolute extension or flexion imbalance. Opposite‐direction associations of ΔaHKA and |ΔaHKA| with final flexion laxity range were observed, suggesting a direction‐sensitive relationship.

**Table 2:** Multivariable linear regression models for associations between continuous radiographic change variables and final intraoperative balance outcomes in the overall cohort.

	Final extension imbalance		Final flexion imbalance		Final extension laxity range		Final flexion laxity range	
Variable	*β* (standardised *β*)	*p*	*β* (standardised *β*)	*p*	*β* (standardised *β*)	*p*	*β* (standardised *β*)	*p*
ΔaHKA	0.024 (0.099)	0.218	−0.010 (−0.030)	0.616	−0.017 (−0.051)	0.322	0.101 (0.124)	<0.001
|ΔaHKA|	0.018 (0.049)	0.582	−0.007 (−0.015)	0.824	0.005 (0.011)	0.839	−0.143 (−0.117)	0.002
ΔJLO	0.020 (0.075)	0.156	−0.060 (−0.182)	<0.001	−0.055 (−0.151)	0.002	−0.046 (−0.053)	0.133
|ΔJLO|	0.025 (0.073)	0.174	−0.046 (−0.106)	0.018	−0.070 (−0.150)	0.004	−0.009 (−0.008)	0.824

*Note*: *β* indicates the regression coefficient. Positive values for aHKA change indicate a more valgus‐directed correction. Final extension and flexion imbalance were defined as the absolute difference between the medial and lateral compartment gaps. Varus–valgus laxity range was defined as the maximum varus angle minus the maximum valgus angle. All models were adjusted for the corresponding initial balance measure, age, sex, body mass index, and implant type.

Abbreviations: aHKA, arithmetic hip–knee–ankle angle; JLO, joint line obliquity.

Using the 2.5‐mm threshold, 71 knees (17.7%) were classified as having final flexion imbalance. Greater ΔJLO was associated with lower odds of final flexion imbalance greater than 2.5 mm (odds ratio, 0.905; 95% CI, 0.844–0.970; *p* = 0.005), as was JLO‐zone CPAK change (odds ratio, 0.487; 95% CI, 0.290–0.817; *p* = 0.006).

In subgroup analyses, JLO redistribution remained the clearest correlate of final flexion balance in varus knees, whereas coronal correction was more closely associated with final flexion laxity range in valgus knees (Table S1).

### Exploratory compartment‐specific resection analyses

In exploratory analyses, ΔJLO was associated with compartment‐specific resection redistribution (Table 3). Greater ΔJLO was associated with greater distal medial femoral resection thickness and lateral tibial resection thickness and with smaller distal lateral femoral resection thickness and medial tibial resection thickness. Greater ΔJLO was also associated with smaller posterior lateral femoral resection thickness, whereas no significant associations were identified with posterior medial femoral resection thickness or tibial construct thickness.

**Table 3:** Exploratory multivariable linear regression models for associations of continuous radiographic change variables with compartment‐specific resection thicknesses and tibial construct thickness in the overall cohort.

	ΔaHKA		|ΔaHKA|		ΔJLO		|ΔJLO|	
Variable	*β*	*p*	*β*	*p*	*β*	*p*	*β*	*p*
Distal medial femoral resection thickness	−0.092	<0.001	0.088	<0.001	0.066	<0.001	0.066	0.003
Distal lateral femoral resection thickness	0.196	<0.001	−0.235	<0.001	−0.110	<0.001	−0.095	0.003
Posterior medial femoral resection thickness	0.034	0.058	−0.034	0.174	0.007	0.684	0.035	0.118
Posterior lateral femoral resection thickness	0.002	0.934	−0.062	0.070	−0.113	<0.001	−0.079	0.007
Medial tibial resection thickness	−0.119	<0.001	0.004	0.901	−0.094	<0.001	−0.091	0.007
Lateral tibial resection thickness	0.225	<0.001	−0.279	<0.001	0.090	<0.001	0.092	0.006
Tibial construct thickness	−0.021	0.093	0.044	0.026	0.005	0.613	0.011	0.375

*Note*: *β* indicates the regression coefficient. Distal medial and lateral femoral resection thicknesses, posterior medial and lateral femoral resection thicknesses, medial and lateral tibial resection thicknesses, and tibial construct thickness were analysed in millimetres. Tibial construct thickness was defined as the combined tibial baseplate and polyethylene thickness used at the final robotic assessment. All models were adjusted for age, sex, body mass index, and implant type.

Abbreviations: aHKA, arithmetic hip–knee–ankle angle; JLO, joint line obliquity.

### Varus‐only tibial‐boundary analysis

Because the tibial varus boundary was most directly relevant to varus knees managed with restricted kinematic alignment, an exploratory varus‐only analysis was performed. Among 207 varus knees, post‐operative tibial varus was matched to native tibial varus within 1° in 41 knees (19.8%), an intermediate change was observed in 34 knees (16.4%), and tibial varus was reduced by more than 2° from native in 132 knees (63.8%). Compared with native‐matched knees, greater |ΔJLO| (5.7 ± 3.3° vs. 1.5 ± 1.1°; *β* = 4.227; *p* < 0.001), greater |ΔaHKA| (3.2 ± 2.3° vs. 1.4 ± 0.9°; *β* = 1.872; *p* < 0.001) and higher odds of overall CPAK change (odds ratio, 4.518; *p* < 0.001) were observed in tibial‐varus‐reduction knees (Table S2). In continuous models, each 1° of tibial‐varus reduction was associated with greater change in LDFA (*β* = 0.386; *p* < 0.001), greater distal femoral resection asymmetry (*β* = 0.277; *p* < 0.001) and smaller final flexion imbalance (*β* = −0.135; *p* = 0.001). After the coronal femoral variables were added to the model, the association with final flexion imbalance was attenuated (*β* = −0.079; *p* = 0.044).

### Patient‐reported outcomes

Among 304 knees from 264 patients with available KOOS‐JR data, the mean KOOS‐JR score improved from 51.3 ± 13.4 preoperatively to 79.4 ± 16.0 post‐operatively, with a mean improvement of 28.1 ± 18.0 points; 229 knees (75.3%) achieved the 16‐point MCID threshold. Neither |ΔaHKA| nor |ΔJLO| was significantly associated with KOOS‐JR improvement or MCID achievement after adjustment for absolute preoperative aHKA, KL grade, age, sex, BMI and implant type (Table S3).

## DISCUSSION

The most important finding of the present study was that greater change in JLO was associated with smaller final flexion imbalance and a narrower final extension laxity range after robot‐assisted TKA, whereas aHKA change showed less consistent associations with final balance. These findings provide a clinically relevant perspective on post‐operative phenotype change during functional alignment. Rather than treating preservation of the native JLO or CPAK phenotype as an obligatory balancing objective, surgeons may interpret post‐operative coronal phenotype together with the quantitative gap and laxity targets achieved during robotic balancing. Within this workflow, modification of JLO did not necessarily represent compromised balance; greater JLO change was observed in knees with smaller final flexion imbalance and a narrower final extension laxity range. Accordingly, modification of JLO within predefined alignment boundaries should not, by itself, be regarded as evidence of unsuccessful balancing. This distinction is particularly relevant to functional alignment, in which robotic TKA allows surgeons to integrate radiographic alignment with real‐time quantitative assessment of ligament balance.

Although the coefficients were numerically small when expressed per 1° of radiographic change, the standardised *β* coefficients for the associations of ΔJLO with final flexion imbalance and final extension laxity range were −0.182 and −0.151, respectively. A 5° increase in ΔJLO corresponded to a 0.301‐mm smaller final flexion imbalance and a 0.273° narrower final extension laxity range. These effect sizes were modest at the individual‐knee level; however, their consistency across two complementary balance outcomes indicates that the observed association was not confined to a single balance metric.

One possible explanation for the more consistent associations observed for JLO is that JLO is derived from the combined coronal orientation of the distal femur and proximal tibia and may therefore represent reorientation at the joint surface more directly than aHKA, for which similar changes may result from different combinations of femoral and tibial adjustment. Only weak relationships between CPAK, aHKA or JLO and preoperative tibiofemoral gaps were found in a recent cohort [14], and patient‐specific laxity phenotype patterns were altered by bony alignment decisions in a simulation study [12]. Together with earlier work on JLO, alignment strategy and quantitative balance [6, 7, 22, 23, 30], these findings support the separation of bony phenotype from soft‐tissue behaviour.

The deformity‐specific analyses provided further support. In varus knees, the clearest association was identified between JLO‐related CPAK change and final flexion balance, whereas in valgus knees, aHKA change was more closely related to final flexion laxity range. Distinct implant‐positioning and resection adjustments in varus and valgus knees have also been reported under robotic functional alignment [18]. Because different alignment strategies were used for varus and valgus knees in the present cohort [27, 29], the subgroup findings should be regarded as supportive rather than definitive.

The exploratory compartment‐specific resection analyses provided additional context for the observed associations. Greater JLO change was associated with redistribution of distal femoral and tibial resection thicknesses, but not with tibial construct thickness. The absence of an association with tibial construct thickness suggests that the observed JLO associations were not explained solely by the thickness of the baseplate–polyethylene construct used at the final robotic assessment. In a prospective robotic anatomo‐functional alignment series, JLO preservation and gap targets were achieved in most knees [9]; the present findings extend that evidence by showing that JLO change can also coexist with a balanced final intraoperative state.

In the exploratory varus‐only analysis, tibial‐varus reduction was associated with greater JLO and CPAK redistribution and smaller final flexion imbalance. Changes in LDFA and distal femoral resection asymmetry were also associated with tibial‐varus reduction, and its association with final flexion imbalance was attenuated after these femoral variables were added to the model. The combined pattern of associations was consistent with coordinated femoral–tibial redistribution within the predefined alignment boundaries rather than isolated tibial correction. This pattern is consistent with the distinct femoral and tibial resection strategies recently reported across varus and valgus knees [18].

Conflicting findings have been reported regarding the clinical relevance of CPAK or JLO preservation. Preservation of native CPAK characteristics or JLO has been associated with better clinical outcomes in some studies [1, 8, 16, 17], whereas no clear adverse effect of post‐operative CPAK change on patient‐reported outcomes has been identified in others [4, 5]. In a randomised trial, an overall advantage of robotic functional alignment over mechanical alignment was not demonstrated, although a possible benefit was observed in constitutionally varus knees classified as CPAK Type I [31]. These mixed findings may reflect phenotype‐specific responses to alignment correction and differences in the balance ultimately achieved.

No significant association between absolute aHKA or JLO change and KOOS‐JR improvement or MCID achievement was identified in the exploratory patient‐reported outcome analyses. This negative finding indicates that the observed intraoperative associations should not be interpreted as evidence of patient benefit. It is consistent with a recent robot‐assisted TKA study in which bone recuts improved intraoperative ligament balance, although the achieved balance was not associated with superior clinical outcomes at 2 years [2]. Post‐operative change in JLO should therefore be interpreted together with the final gap and laxity measurements, rather than as an isolated indicator of technical error or clinical benefit. Prospective studies directly linking standardised intraoperative balancing parameters with long‐term PROMs are needed to determine whether the observed relationships between radiographic change and intraoperative balance translate into clinically meaningful improvements in patient‐reported outcomes.

Several limitations should be acknowledged. First, this was a retrospective, single‐surgeon study using a single robotic platform, and no external validation cohort was available. Although this provided a consistent operative and measurement workflow, the generalisability of the findings to other surgeons, robotic systems, stress techniques and alignment protocols may be limited. Importantly, the alignment philosophy differed according to the preoperative deformity pattern: varus knees were generally treated using a restricted kinematic alignment strategy, whereas valgus knees were treated using a femoral‐referenced, slight‐valgus mechanical alignment strategy. Consequently, differences observed between varus and valgus knees may reflect both the underlying deformity phenotype and strategy‐specific planning and balancing decisions. These effects could not be separated in the present study. Component positioning and balancing adjustments were also selected in response to intraoperative findings rather than being randomly assigned, introducing the possibility of confounding by indication. Accordingly, the specific associations between resection patterns, radiographic changes and final balance observed in this cohort may partly reflect this surgeon‐specific operative workflow and should not be assumed to represent a generalisable balancing sequence. Second, selection bias cannot be excluded. Among the 446 otherwise eligible knees, the 402 included knees and 44 knees excluded because of incomplete paired robotic measurements were generally similar in BMI, implant type, preoperative HKA‐defined deformity distribution and KOOS‐JR improvement (all absolute standardised mean differences < 0.10), although included knees showed slightly greater mean preoperative varus (aHKA, −1.3° vs. 0.0°; standardised mean difference, 0.213). Nevertheless, inclusion in the main analysis required both preoperative and post‐operative full‐length radiographs and complete paired initial and final intraoperative balance measurements, and the availability of these data may not have been random. Patient‐reported outcomes were available only in a subset of the cohort, further increasing the possibility of selection bias and limiting the statistical power and generalisability of the KOOS‐JR analyses. Clinical follow‐up was limited to the 1‐year KOOS‐JR assessment, and implant survivorship was not evaluated. Third, residual and unmeasured confounding may persist despite adjustment for the corresponding initial balance or laxity measure and relevant patient and implant characteristics. In particular, PCL management varied according to intraoperative balancing requirements: CR and PS implants were both used, and the PCL was selectively released when required in CR knees. Although implant type was included in the multivariable models, the extent of PCL release was not separately quantified and may have influenced the final flexion balance measurements. The radiographic changes and final balance measurements also resulted from the same iterative planning and balancing process; therefore, the observed associations cannot establish causality or directionality. In particular, the association between greater JLO change and smaller final flexion imbalance should not be interpreted as evidence that targeting a greater change in JLO would improve balance. Fourth, the initial and final intraoperative measurements were obtained by the same surgeon using manually applied stress without an instrumented device or a predefined force magnitude. Although a consistent technique was used and a recent in vivo study using the ROSA system reported good‐to‐excellent intra‐ and interoperator reproducibility for soft‐tissue laxity and gap assessments under manual stress [11], intraobserver reproducibility was not evaluated in the present cohort, and variation in the magnitude and direction of stress may have influenced the recorded gaps and laxity ranges. Moreover, the final measurements were obtained intraoperatively with trial components and may not directly represent post‐operative or weight‐bearing functional stability. Radiographic measurements may also have been affected by limb positioning and rotational variation, despite the good‐to‐excellent interobserver reliability observed in this study. Fifth, the varus‐only tibial‐boundary analysis and the other subgroup and secondary analyses, including those of compartment‐specific resection thickness and coronal femoral adjustment, were exploratory and hypothesis‐generating. They should not be interpreted as definitive evidence of the surgical mechanisms underlying the observed associations. Detailed information regarding the extent and sequence of soft‐tissue releases was not consistently available, preventing comprehensive assessment of their contribution to the final balance state. These findings require confirmation in independent cohorts. No formal adjustment for multiple comparisons was applied, and the number of regression models increases the possibility of type I error. Individual findings, particularly those from the secondary, subgroup, and resection‐related analyses, should therefore be interpreted cautiously and require confirmation in independent cohorts. Finally, this study was not designed to compare competing alignment concepts or to determine whether a patient's native CPAK phenotype should be preserved or changed. Accordingly, the findings should not be interpreted as demonstrating the clinical superiority of a particular post‐operative alignment target or CPAK transition. Prospective multicentre studies using standardised stress application, predefined alignment strategies and more complete longitudinal clinical outcomes are required to determine the clinical implications of these radiographic and intraoperative relationships.

## CONCLUSION

Within an individualised robot‐assisted TKA alignment and balancing workflow, greater JLO change was associated with smaller final flexion imbalance and a narrower extension laxity range. In varus knees, JLO change was accompanied by tibial‐varus reduction and coordinated coronal femoral adjustment. CPAK change involving JLO may therefore reflect aspects of the osseous and soft‐tissue adjustments accompanying the final intraoperative balancing state rather than unintended deviation from the native phenotype.

## AUTHOR CONTRIBUTIONS

**Yugo Morita**: Conceptualisation; data curation; formal analysis; methodology; writing—original draft. **Jonggu Shin**: Investigation; validation; writing—review and editing. **Mohammed El‐Hassan**: Data curation; writing—review and editing. **Ittai Shichman**: Writing—review and editing. **William J. Long**: Writing—review and editing. **Peter K. Sculco**: Conceptualisation; project administration; resources; supervision; writing—review and editing.

## FUNDING INFORMATION

The authors have no funding to report.

## CONFLICT OF INTEREST STATEMENT

William J. Long reports royalties from Zimmer Biomet and Elsevier; speakers bureau and paid presentations for Zimmer Biomet, Sylke and Convatec; stock or stock options in Sylke; service on the editorial or governing board of the *Journal of Arthroplasty*; and board or committee appointments with the Hip Society, the Knee Society and the American Joint Registry Steering Committee. Peter K. Sculco reports royalties from Enovis; speakers bureau/paid presentations for Zimmer; paid consultancy for Enovis and Zimmer; stock or stock options in Intellijoint Surgical and Parvizi Surgical Innovation; research support from Intellijoint Surgical as a principal investigator; and a board or committee appointment with *HSS Journal*. The other authors declare no conflicts of interest.

## ETHICS STATEMENT

Institutional review board approval (IRB 2025‐0624) was obtained from the participating institution. Informed consent was waived owing to the retrospective nature of the study.

## Supporting information

Supporting File 1:

## Data Availability

The data that support the findings of this study are available on request from the corresponding author. The data are not publicly available due to privacy or ethical restrictions.
