# Supplementary material for: Joint line obliquity change was associated with femoral and tibial resection patterns and smaller final flexion imbalance after robot‐assisted total knee arthroplasty
Source: J Exp Orthop. 2026 Oct 3;13(4):e70936. doi: 10.1002/jeo2.70936 (PMC13633672; doi:10.1002/jeo2.70936)
Supplement: Supplementary file 1 [file JEO2-13-e70936-s001.docx]

**Supplementary Table 1.** Multivariable linear regression models for associations between continuous radiographic change variables and final intraoperative balance outcomes in varus and valgus knees

| Variable | Final extension imbalance | | Final flexion imbalance | | Final extension  laxity range | | Final flexion  laxity range | |
| --- | --- | --- | --- | --- | --- | --- | --- | --- |
|  | β | *p* value | β | *p* value | β | *p* value | β | *p* value |
| Varus knees | |  |  |  |  |  |  |  |
| ΔaHKA | 0.056 | 0.042 | -0.036 | 0.225 | -0.030 | 0.559 | -0.002 | 0.968 |
| \|ΔaHKA\| | 0.056 | 0.107 | -0.073 | 0.094 | -0.002 | 0.975 | 0.019 | 0.777 |
| ΔJLO | 0.006 | 0.757 | -0.086 | 0.001 | -0.068 | 0.018 | -0.065 | 0.086 |
| \|ΔJLO\| | 0.007 | 0.765 | -0.064 | 0.012 | -0.079 | 0.040 | -0.029 | 0.554 |
| Valgus knees | |  |  |  |  |  |  |  |
| ΔaHKA | -0.027 | 0.618 | 0.003 | 0.954 | 0.023 | 0.528 | 0.160 | 0.017 |
| \|ΔaHKA\| | 0.038 | 0.490 | 0.002 | 0.972 | -0.021 | 0.593 | -0.161 | 0.024 |
| ΔJLO | 0.054 | 0.114 | -0.046 | 0.304 | 0.005 | 0.890 | -0.011 | 0.890 |
| \|ΔJLO\| | 0.073 | 0.128 | -0.005 | 0.920 | 0.001 | 0.986 | 0.127 | 0.235 |

β indicates the regression coefficient. Final extension and flexion imbalance were each defined as the absolute difference between the medial and lateral compartment gaps. Varus–valgus laxity range was defined as the maximum varus angle minus the maximum valgus angle. Radiographic change variables and laxity ranges were expressed in degrees, and imbalance was expressed in millimetres. All models were adjusted for the corresponding initial balance measure, age, sex, body mass index and implant type. Varus knees were defined by a preoperative aHKA less than −2°, and valgus knees by a preoperative aHKA greater than 2°.

aHKA, arithmetic hip–knee–ankle angle; JLO, joint line obliquity.

**Supplementary Table 2.** Varus-only tibial-boundary analysis and coronal femoral adjustment

| Variable | Tibial-varus reduction >2° vs native-matched | | Per 1° tibial-varus reduction | |
| --- | --- | --- | --- | --- |
|  | β or OR | *p* value | β or OR | *p* value |
| \|ΔJLO\| | 4.227 | < 0.001 | 0.888 | < 0.001 |
| \|ΔaHKA\| | 1.872 | < 0.001 | 0.435 | < 0.001 |
| Overall CPAK change | 4.518 | < 0.001 | 1.323 | < 0.001 |
| Change in LDFA | 1.687 | < 0.001 | 0.386 | < 0.001 |
| Distal femoral resection asymmetry | 1.027 | 0.002 | 0.277 | < 0.001 |
| Final flexion imbalance | -0.531 | 0.040 | -0.135 | 0.001 |
| Final flexion imbalance after adding coronal femoral variables | — | — | -0.079 | 0.044 |

Values are shown as β coefficients or odds ratios, with p values. Overall CPAK change is shown as an OR; all other estimates are β coefficients. Native-matched knees were defined as knees in which postoperative tibial varus was restored within 1° of native tibial varus; tibial-varus-reduction knees were defined as knees in which postoperative tibial varus was reduced by > 2° from native. The direct comparison contrasts tibial-varus-reduction knees with native-matched knees. The continuous model evaluates tibial-varus reduction from native as a continuous variable per 1°. Angular outcomes were expressed in degrees; distal femoral resection asymmetry and final flexion imbalance were expressed in millimetres. Models were adjusted for age, sex, body mass index, implant type and KL grade, with patient-level cluster-robust standard errors. Final flexion imbalance models were additionally adjusted for initial flexion imbalance. The attenuation model additionally included change in LDFA and distal femoral resection asymmetry.

aHKA, arithmetic hip–knee–ankle angle; CPAK, Coronal Plane Alignment of the Knee; JLO, joint line obliquity; KL, Kellgren–Lawrence; LDFA, lateral distal femoral angle; OR, odds ratio.

**Supplementary Table 3.** Exploratory multivariable linear and logistic regression models for associations between absolute radiographic change variables and KOOS-JR outcomes in the available subset

|  |  | \|ΔaHKA\| | |  | | \|ΔJLO\| | |  | |
| --- | --- | --- | --- | --- | --- | --- | --- | --- | --- |
| Variable | Mean (range) or n/N (%) | β or OR | *p* value | | β or OR | | *p* value | |  |
| Preoperative KOOS-JR | 51.3 ± 13.4 (0.0 to 84.6) | — | — | | — | | — | |  |
| Postoperative KOOS-JR | 79.4 ± 16.0 (20.9 to 100.0) | — | — | | — | | — | |  |
| Change in KOOS-JR | 28.1 ± 18.0 (−34.6 to 92.0) | -0.246 | 0.606 | | -0.292 | | 0.339 | |  |
| MCID achievement | 229/304 (75.3%) | 0.937 | 0.329 | | 0.986 | | 0.755 | |  |

Values are presented as mean ± SD (range) or n/N (%). Change in KOOS-JR was calculated as postoperative minus preoperative score. Achievement of the MCID was defined as an improvement of at least 11.5 points. β coefficients are shown for linear regression models evaluating change in KOOS-JR, and odds ratios are shown for logistic regression models evaluating MCID achievement. Models were adjusted for absolute preoperative aHKA, KL grade, age, sex, body mass index and implant type, with patient-level cluster-robust standard errors.

aHKA, arithmetic hip–knee–ankle angle; JLO, joint line obliquity; KL, Kellgren–Lawrence; KOOS-JR, Knee injury and Osteoarthritis Outcome Score for Joint Replacement; MCID, minimal clinically important difference; OR, odds ratio; SD, standard deviation.

**Supplementary Table 4.** Transitions between preoperative and postoperative CPAK phenotypes

| Preoperative CPAK type | Type I | Type II | Type III | Type IV | Type V | Type VI | Type VII | Type VIII | Type IX |
| --- | --- | --- | --- | --- | --- | --- | --- | --- | --- |
| Type I | 44 (30.6%) | 7 (4.9%) | 0 (0.0%) | 63 (43.8%) | 22 (15.3%) | 1 (0.7%) | 3 (2.1%) | 4 (2.8%) | 0 (0.0%) |
| Type II | 9 (13.2%) | 17 (25.0%) | 3 (4.4%) | 12 (17.6%) | 24 (35.3%) | 2 (2.9%) | 0 (0.0%) | 1 (1.5%) | 0 (0.0%) |
| Type III | 4 (4.8%) | 15 (17.9%) | 4 (4.8%) | 12 (14.3%) | 37 (44.0%) | 11 (13.1%) | 0 (0.0%) | 1 (1.2%) | 0 (0.0%) |
| Type IV | 8 (19.5%) | 2 (4.9%) | 0 (0.0%) | 20 (48.8%) | 9 (22.0%) | 0 (0.0%) | 1 (2.4%) | 1 (2.4%) | 0 (0.0%) |
| Type V | 5 (20.8%) | 0 (0.0%) | 0 (0.0%) | 6 (25.0%) | 12 (50.0%) | 1 (4.2%) | 0 (0.0%) | 0 (0.0%) | 0 (0.0%) |
| Type VI | 0 (0.0%) | 6 (16.2%) | 1 (2.7%) | 4 (10.8%) | 21 (56.8%) | 5 (13.5%) | 0 (0.0%) | 0 (0.0%) | 0 (0.0%) |
| Type VII | 0 (0.0%) | 0 (0.0%) | 0 (0.0%) | 3 (100.0%) | 0 (0.0%) | 0 (0.0%) | 0 (0.0%) | 0 (0.0%) | 0 (0.0%) |
| Type VIII | 0 (0.0%) | 0 (0.0%) | 0 (0.0%) | 1 (100.0%) | 0 (0.0%) | 0 (0.0%) | 0 (0.0%) | 0 (0.0%) | 0 (0.0%) |
| Type IX | 0 (0.0%) | 0 (0.0%) | 0 (0.0%) | 0 (0.0%) | 0 (0.0%) | 0 (0.0%) | 0 (0.0%) | 0 (0.0%) | 0 (0.0%) |

Values are presented as n (percentage within each preoperative CPAK phenotype). Rows represent preoperative phenotypes and columns represent postoperative phenotypes. Diagonal cells indicate preservation of the same CPAK phenotype. Percentages may not total 100.0% because of rounding.

CPAK, Coronal Plane Alignment of the Knee.
